# 

**Published:** 1891-06

**Authors:** 


### CONTENTS.

### COMMUNICATIONS.

The History of Dentistry as a Co-ordinate Branch of the Healing Art .......... 265
Development of Teeth .......... 270
Recurrence of Decay .......... 281--283
The Lecture and the Lesson .......... 285
A Dentist and La Grippe .......... 289
Translation—Stomatitis .......... 291

### SELECTIONS.

Odontological Society of England .......... 295
Dietetic Progress .......... 301
Hypnotism in Public Forbidden .......... 301
Constituents of Food .......... 302
Why Carbolic Acid should be discarded by Dentists .......... 302
Oat Meal .......... 303
Mummies .......... 303
Rights and Lefts .......... 304
Food Products and their Cheif Adulterants .......... 305
Cures for Neuralgia of the Head .......... 305

### CORRESPONDENCE.

A Communication from the Dental Protective Association of the United States .......... 306

### SOCIETIES.

National Association of Boards of Dental Examiners .......... 308
National Association of Dental Faculties .......... 308
Missouri State Dental Association .......... 309

### EDITORIAL.

Dental Offices .......... 311
Michigan Meeting .......... 313
Obituary .......... 314

